# Impact of out-of-home care on children’s outcomes: A longitudinal cohort study using linked administrative data from Manitoba, Canada

**DOI:** 10.23889/ijpds.v9i5.1679

**Published:** 2024-09-18

**Authors:** Marni Brownell, Kayla Frank, Nathan Nickel, Jennifer Enns, Lisa Flaten, Stephanie Sinclair, Elizabeth Decaire, Jennifer Chartrand, Scott Sinclair, Teresa Mayer, Chris Nash, Jamie Pfau, Anita Durksen, Colette Scatliff, Mikayla Hunter, Soomin Han, Stephaney Patrick, Hera Casidsid, Sana Amjad, Marlyn Bennett, Hygiea Casiano, James Houston, Laura Bowler, Therese Stukel

**Affiliations:** 1 University of Manitoba; 2 Assembly of Manitoba Chiefs; 3 First Nations Health and Social Secretariat of Manitoba; 4 Government of Manitoba; 5 McGill University; 6 University of Calgary; 7 University of Toronto

## Introduction

To provide rigorous quantitative evidence about the impact of out-of-home care on children’s outcomes.

## Approach

Guided by an Advisory Circle of First Nations (FN) Knowledge Keepers and conducted in partnership with FN researchers, we used linked administrative data to identify all Manitoba children served by Child Protection Services (CPS) at FN agencies (FNA) and other agencies (OA) (2007-2018). We compared health, education, and legal system outcomes of those in care (FNA n=10,856; OA n=8,468) to those with protection concerns but not in care (FNA n=12,896; OA n=14,394). Using instrumental variable analysis with CPS agency rate of out-of-home care as the instrument, outcomes between groups were compared using 2-stage multivariate probit regressions adjusted for child and family factors.

## Results

Odds of teen pregnancy (FNA OR 3.69, 95% CI 1.40-9.77; OA 5.10 (1.83-14.25)), teen birth (FNA 3.23 (1.10-9.49); OA 5.06 (1.70-15.03)), and positive STI tests (FNA ns; OA 7.21 (3.63-14.32)) were higher and odds of vaccination at age 2 (FNA ns; OA 0.49 (0.29-0.80)) were lower for children in care than children not in care. Odds of being accused (FNA ns; OA 2.71 (1.27-5.75)), victim (FNA ns; OA 1.68 (1.10-2.56)), or charged with a crime (FNA ns; OA 2.68 (1.21-5.96)), and odds of being incarcerated (FNA 3.64 (1.95-6.80); OA 1.19 (1.19-8.04)) were higher for children in care.

## Conclusions

Out-of-home care negatively affects children’s health, social and legal system outcomes.

## Implications

CPS agencies should work with FN leaders to develop and implement strategies to reduce the number of children taken into care.

